# Antioxidant Effects of Oral Ang-(1-7) Restore Insulin Pathway and RAS Components Ameliorating Cardiometabolic Disturbances in Rats

**DOI:** 10.1155/2019/5868935

**Published:** 2019-07-14

**Authors:** Vivian Paulino Figueiredo, Maria Andrea Barbosa, Uberdan Guilherme Mendes de Castro, Aline Cruz Zacarias, Frank Silva Bezerra, Renata Guerra de Sá, Wanderson Geraldo de Lima, Robson Augusto Souza dos Santos, Andréia Carvalho Alzamora

**Affiliations:** ^1^NUPEP, Universidade Federal de Ouro Preto, Ouro Preto, MG, Brazil; ^2^Departamento de Ciências Biológicas, Instituto de Ciências Exatas e Biológicas, Brazil; ^3^Departamento de Fisiologia e Biofísica, Universidade Federal de Minas Gerais, Belo Horizonte, MG, Brazil

## Abstract

In prevention studies of metabolic syndrome (MetS), Ang-(1-7) has shown to improve the insulin signaling. We evaluated the HP*β*CD/Ang-(1-7) treatment on lipid metabolism, renin-angiotensin system (RAS) components, oxidative stress, and insulin pathway in the liver and gastrocnemius muscle and hepatic steatosis in rats with established MetS. After 7 weeks of high-fat (FAT) or control (CT) diets, rats were treated with cyclodextrin (HP*β*CD) or HP*β*CD/Ang-(1-7) in the last 6 weeks. FAT-HP*β*CD/empty rats showed increased adiposity index and body mass, gene expression of ACE/ANG II/AT1R axis, and oxidative stress. These results were accompanied by imbalances in the insulin pathway, worsening of liver function, hyperglycemia, and dyslipidemia. Oral HP*β*CD/Ang-(1-7) treatment decreased *ACE* and *AT1R*, increased *ACE2* gene expression in the liver, and restored thiobarbituric acid reactive substances (TBARS), catalase (CAT), superoxide dismutase (SOD), insulin receptor substrate (*Irs-1*), glucose transporter type 4 (*GLUT4*), and serine/threonine kinase 2 (*AKT-2*) gene expression in the liver and gastrocnemius muscle improving hepatic function, cholesterol levels, and hyperglycemia in MetS rats. Overall, HP*β*CD/Ang-(1-7) treatment restored the RAS components, oxidative stress, and insulin signaling in the liver and gastrocnemius muscle contributing to the establishment of blood glucose and lipid homeostasis in MetS rats.

## 1. Introduction

The angiotensin-converting enzyme (ACE)2/angiotensin-(1-7)/Mas receptor axis belongs to the renin-angiotensin system (RAS) and has antiproliferative, antioxidant, and anti-inflammatory properties. This axis induces beneficial effects in hypertension, glucose intolerance, and insulin resistance (IR), and it acts in a counter-regulatory way to another important RAS axis, ACE/Ang II/AT1 receptor [1]. Imbalances in the actions of RAS components can trigger many pathological processes and disturbances in the metabolic functions of the liver and muscle, such as those observed in the metabolic syndrome (MetS) and diabetes mellitus type 2 (DM2) [2–4]. Previous studies have shown the correlation between increased oxidative stress and IR [5, 6] and hyperactivity of the ACE/Ang II/AT1 axis [7, 8]. Increased oxidative stress impairs glucose uptake in the muscle and in the liver, decreasing insulin secretion of pancreatic *β* cells in DM2 and MetS states [5, 6, 9].

A high-fat (FAT) diet has been reported as risk factor RAS components for the development of DM2 and MetS [10, 11]. Ang-(1-7) prevents the damage in the insulin pathway in the gastrocnemius muscle and liver in the development of MetS [10, 12]. However, there are no studies on the effect of an oral Ang-(1-7) treatment on the established MetS correlating oxidative stress, insulin signaling pathway, and RAS components balance. Thus, we evaluated whether Ang-(1-7) included in the oligosaccharide hydroxypropyl-*β*-cyclodextrin (HP*β*CD) can be used as treatment in MetS already established in rats fed with FAT diet on lipid homeostasis, RAS axis components, oxidative stress, insulin pathway, and liver function. HP*β*CD has been reported to protect the Ang-(1-7) peptide during passage through the gastrointestinal tract after oral administration [13, 14].

## 2. Materials and Methods

### 2.1. Ethical Approval

All procedures were performed in accordance with the Guidelines for Ethics in Care of Experimental Animals. The project was approved by the animal ethics committee of the Federal University of Ouro Preto protocol 2011/31.

### 2.2. Study Design

Four-week-old Fisher rats, weighing 49 ± 7 g (*n* = 28), from the animal facility of UFOP remained in individual cages (22 ± 2°C) with a light-dark cycle from 12 h to 12 h and free access to water and diets. After weaning, the animals were fed with control diet (AIN-93M, CT: carbohydrate 75,82%; fat 9,46%; protein 14,72%; kcal/g 3,8) or high-fat diet [FAT: carbohydrate 15,92%; fat 68,48%-37% from lard; protein 15,60%; kcal/g 5,2] for 13 weeks, and food intake was evaluated weekly. In the seventh week of diets, MetS symptoms were analyzed such as body mass, fasting (12 h) glucose using commercial kits (Labtest, Lagoa Santa, MG, Brazil), and mean arterial pressure (MAP) and heart rate (HR) by digital tail plethysmography (Panlab, LE5001). After seven weeks of the diets, orally by gavage, the treatment with HP*β*CD/Ang-(1-7) (40 *μ*g/kg/day) or HP*β*CD/empty [HP*β*CD without the inclusion of Ang-(1-7)] was started for six weeks. At the end of the 13 weeks, the rats were fasted for 12 h and decapitated [6, 11]. Blood samples were collected and centrifuged (8000g, 4°C, 6 min). The serum was collected and the liver, fat deposits (retroperitoneal, epididymal, and inguinal), and gastrocnemius muscle were removed, weighed (g/100 g rat mass), placed in liquid nitrogen, and stored at -80°C for qRT-PCR and oxidative stress evaluations. The experimental groups were as follows: (1) CT-HP*β*CD/empty (*n* = 7): rats fed with CT diet and treated with empty HP*β*CD during the last six weeks of diet; (2) CT-HP*β*CD/Ang-(1-7) (*n* = 7): rats fed with CT diet and treated with HP*β*CD/Ang-(1-7) during the last six weeks of diet; (3) FAT-HP*β*CD/empty (*n* = 7): rats fed with FAT diet and treated with empty HP*β*CD during the last six weeks of diet; and (4) FAT-HP*β*CD/Ang-(1-7) (*n* = 7): rats fed with FAT diet and treated with HP*β*CD/Ang-(1-7) during the last six weeks of diet.

### 2.3. Adiposity Index

Adiposity index was measured by the formula [inguinal fat + epididymal fat + retroperitoneal fat absolute] [15].

### 2.4. Fasting Glucose and Serum Lipid Profile Analysis

At the end of the experiment, after euthanasia by instant decapitation of animals fasted overnight, blood samples (2 to 3 mL) were collected and centrifuged (8000g, 4°C, 6 min) to separate the serum for determination of fasting glucose, total cholesterol, LDL, high-density lipoprotein (HDL), alanine aminotransferase (ALT), and aspartate aminotransferase (AST). The serum was aliquoted and stored at (−80°C) to conduct the biochemical analyses. The analyses were performed using individual commercial kits (Labtest, Lagoa Santa, MG, Brazil) according to the instructions provided by the manufacturer. Analyses were performed in the Pilot Laboratory of Clinical Analyses (LAPAC/UFOP).

### 2.5. Activity of Superoxide Dismutase and Catalase

Frozen liver and gastrocnemius muscle samples (100 mg) were homogenized in phosphate buffer (pH 7.4) and centrifuged at 12.000xg for 10 min at 4°C. Elisa reader at 570 nm was used to determine SOD activity as previously described [6, 16]. CAT activity was measured by the rate of decrease of the hydrogen peroxide (H_2_O_2_) at 240 nm. The total protein content in samples of organ homogenates was determined by using the Bradford method [17]. All results were expressed as activity per protein milligram.

### 2.6. Substance Reactive to Thiobarbituric Acid

The formation of TBARS during an acid warming reaction was used for lipid peroxidation index [6]. Frozen samples of the liver and gastrocnemius muscle (100 mg) were homogenized in KPE (potassium phosphate-EDTA) buffer (pH 7.4) and centrifuged (10.000xg, 10 min at 4°C). The supernatant was collected and mixed with 1 mL of 10% trichloroacetic acid and 1 mL of 0.67% thiobarbituric acid. After this, it was heated in a boiling water bath for 30 min. TBARS were determined from the absorbance at 532 nm. Data series were expressed in nmol/mg protein.

### 2.7. Analysis of Gene Expression

RNA extraction and real-time reverse transcription polymerase chain reaction (qRT-PCR) were performed in the hepatic and muscular tissues as previously described [6]. The analyses were performed by relative method of quantification of gene expression (comparative Cq, *Δ*Cq) and expression values were normalized for the amount of the reference gene (18S rRNA) in each plate. The results were obtained from the formula given by (2-*Δ*Cq). The primer pairs were *Rn18s* (5′-GTAAGTGCGGGTCATAAG-3′ and 5′-CCATCCAATCGGTAGTAGC-3′), *Inrs* (5′-CCTTGGATCGTTCCTCTCAC-3′ and 5′-GGTCCGTTTGATGCTCAGAG-3′), *Irs-1* (5′-TGAGAGCGGTGGTGGTAAGC-3′ and 5′-GGGCTGCTGGTGTTGGAATC-3′), *Irs-2* (5′-GCAGGACTTTCCCAGTGAACG-3′ and 5′-GCCACACCACATTCGCATG-3′), *Akt-2* (5′-GGAGGTCATGGAGCATCGGTTC-3′ and 5′-GTTTGAAGGGTGGCAGGAGC-3′), *Slc2a4* (*Glut-4*) (5′-GGTGCCTTGGGAACACTCAAC-3′ and 5′-TGCAGGAGAGCAGGGAGTACTG-3′), *angiotensinogen* (5′-CTGTGAAGGAGGGAGACTGC-3′ and 5′-CAGCAAGCCCTGACCAGC-3′), A*CE2* (5′-GAGATGAAGCGGGAGATCG-3′ and 5′-TGGAACAGAGATGCAGGGTC-3′), *Agtr1a/b* (5′-TCACTTTCCTGGATGTGCTG-3′ and 5′-GATGGGCATGGCAGTGTC-3′), *Mas* (5′-CAATCGTGACGTTATCGGTG-3′ and 5′-TCTCTCCACACTGAT GGCTG-3′), and *ACE* (5′-TGGCACTTGTCTGTCACTGG-3′ and 5′-ACACCCAAAGCA ATTCTTCG-3′).

### 2.8. Histological Analyses

Fragments of the liver were fixed in 10% formalin for 72 h, dehydrated, cleaned, and embedded in paraffin. The paraffin blocks were sectioned at 4 *μ*m thick and stained with hematoxylin and eosin (H&E). The presence or absence of areas with nonalcoholic micro and macro steatoses (NAFLD) was observed by light microscopy in the hepatic tissue. Representative photomicrographs were obtained with a Leica BM5000 microscope coupled to a Leica DFC 300 FX camera in RGB mode, using a 40x magnification lens.

### 2.9. Statistical Analysis

Results are expressed as means ± SEM. Data were analyzed for Kolmogorov-Smirnov normality and followed the standard normal distribution. After, they were evaluated by two-way ANOVA, followed by Fisher's LSD post test. Statistical analyses were performed with GraphPad Prism software (version 6.0, San Diego, USA). Statistical significance was set at *p* < 0.05.

## 3. Results

### 3.1. Evaluation of MetS Establishment

In the seventh week of diets, the FAT-HP*β*CD/empty rats showed symptoms of MetS such as increased fasting blood glucose, MAP, HR, and body mass, compared to CT-HP*β*CD/empty rats (Table 1).

### 3.2. Evaluation of Nutritional, Biometric, and Biochemical Parameters

In the last six weeks of diets, FAT-HP*β*CD/empty rats displayed increased body mass, adiposity index, fasting glucose, total cholesterol, LDL, triglycerides, and ALT levels compared to CT-HP*β*CD/empty (Table 2). However, FAT-HP*β*CD/Ang-(1-7) rats improved body mass, adiposity index, fasting glucose, total cholesterol, LDL, triglycerides, and ALT levels compared to the CT-HP*β*CD/empty rats (Table 2). Additionally, FAT-HP*β*CD/empty and FAT-HP*β*CD/Ang-(1-7) rats presented decreased food intake but similar caloric intake compared to CT-HP*β*CD/empty. No difference was found in liver and gastrocnemius muscle, HDL, and AST levels between groups (Table 2).

### 3.3. Evaluation of Oxidative Stress

**Figure 1:**
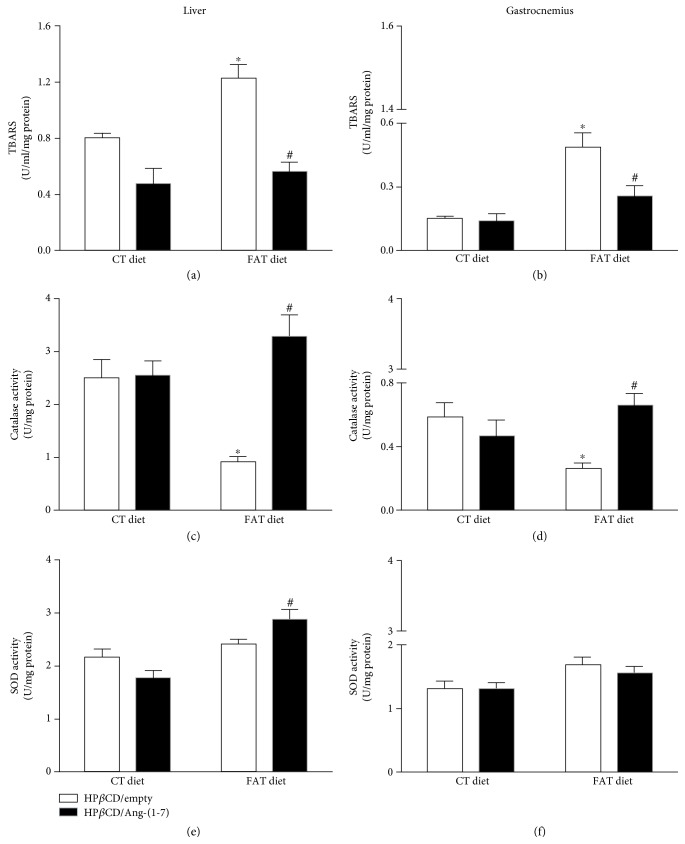
Levels of TBARS (U/mL/mg of protein) (a, b), activity of the catalase enzyme (CAT, U/mg of protein) (c, d), and activity of the enzyme superoxide dismutase (SOD, U/mg of protein) (e, f) in the liver and gastrocnemius muscle of rats subjected to a high-fat diet (37% fat, FAT; *n* = 6) or control diet (AIN-93, CT; *n* = 7) for 13 weeks and treated with vehicle (HP*β*CD) or HP*β*CD-Ang-(1-7) during the last 6 weeks of diets. ^∗^*p* < 0.05 compared to the CT-HP*β*CD/empty group. ^#^*p* < 0.05 compared to the group FAT-HP*β*CD/empty (two-way ANOVA followed by Fisher's LSD posttest).

FAT-HP*β*CD/empty rats presented increased TBARS concentration and decreased CAT activity in the liver and gastrocnemius muscle compared to CT-HP*β*CD/empty rats (Figure 1(a)–(d)). However, FAT-HP*β*CD/Ang-(1-7) rats decreased TBARS concentration and increased CAT activity in the liver and gastrocnemius muscle compared to FAT-HP*β*CD/empty animals (Figure 1(a)–(d)). The concentrations of TBARS and CAT activity in FAT-HP*β*CD/Ang-(1-7) rats became similar to the CT-HP*β*CD/empty rats. In addition, only FAT-HP*β*CD/Ang-(1-7) rats showed increased liver SOD activity compared to FAT-HP*β*CD/empty rats (Figure 1(e)). No difference was observed in SOD activity in the gastrocnemius muscle (Figure 1(f)).

### 3.4. mRNA Expression of the RAS Components and the Insulin Signaling Pathway

**Figure 2:**
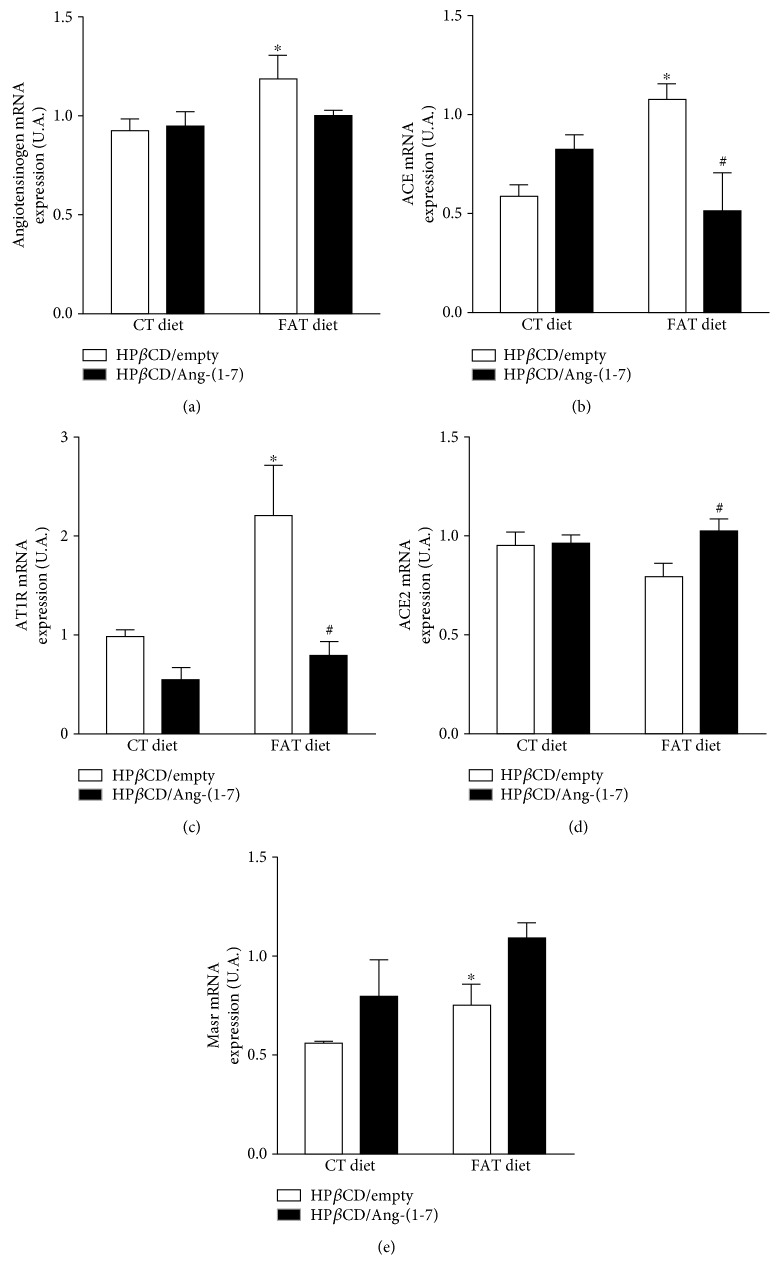
Evaluation of the mRNA expression components of the RAS *angiotensin* (a), angiotensin-converting enzyme (*ACE*) (b), angiotensin II receptor type 1 (*AT1R*) (c), angiotensin-converting enzyme 2 (*ACE2*) (d), and receptor mass (*Masr*) (e) in the liver of rats subjected to a high-fat diet (37% fat, FAT; *n* = 6) or control diet (AIN-93, CT; *n* = 7) for 13 weeks and treated with vehicle (HP*β*CD) or HP*β*CD-Ang-(1-7) during the last 6 weeks of diets. ^∗^*p* < 0.05 compared to the CT-HP*β*CD/empty group. ^#^*p* < 0.05 compared to the group FAT-HP*β*CD/empty (two-way ANOVA followed by Fisher's LSD posttest).

FAT-HP*β*CD/empty rats showed increased *angiotensinogen*, *ACE*, *AT1R*, and *Masr* mRNA gene expression compared to CT-HP*β*CD/empty rats (Figures 2(a)–2(c) and 2(e)). However, FAT-HP*β*CD/Ang-(1-7) rats decreased *ACE* and *AT1R* increasing *ACE2* gene expression compared to FAT-HP*β*CD/empty rats (Figures 2(b)–2(d)).

**Figure 3:**
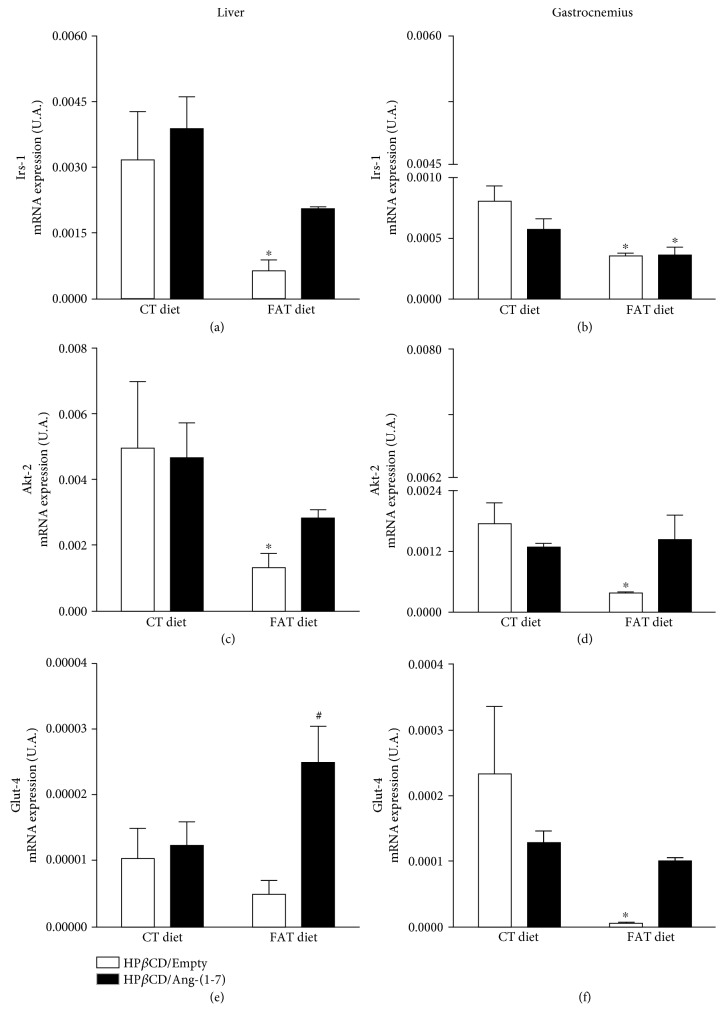
Evaluation of the mRNA expression of insulin signaling pathway mediators. Insulin receptor substrate 1 (*Irs-1*) (a, b), protein kinase B (*Akt-2*) (c, d), and type glucose transporter 4 (*Glut-4*) (e, f) in the liver and gastrocnemius muscle of rats subjected to a high-fat diet (37% fat, FAT; *n* = 6) or control diet (AIN-93, CT; *n* = 7) for 13 weeks and treated with vehicle (HP*β*CD) or HP*β*CD-Ang-(1-7) during the last 6 weeks of diets. ^∗^*p* < 0.05 compared to the CT-HP*β*CD/empty group. ^#^*p* < 0.05 compared to the FAT-HP*β*CD/empty group (two-way ANOVA followed by Fisher's LSD posttest).

FAT-HP*β*CD/empty rats presented lower gene expression of the *Irs-1* and *Akt-2* components of the intracellular insulin pathway in the liver and gastrocnemius muscle compared to CT-HP*β*CD/empty rats (Figure 3(a)–(d)). In addition, FAT-HP*β*CD/empty group presented lower *Glut-4* gene expression in the gastrocnemius muscle compared to CT-HP*β*CD/empty rats (Figure 3(f)). FAT-HP*β*CD/Ang-(1-7) rats presented increased *Glut-4* gene expression in the liver compared to FAT-HP*β*CD/empty group (Figure 3(e)). In addition, in the FAT-HP*β*CD/Ang-(1-7) rats, the mRNA expression of *Glut-4* became similar to CT-HP*β*CD/empty rats in the liver and gastrocnemius muscle (Figures 3(e) and (f)). No differences in mRNA expression of *Inrs* and *Irs-2* were observed among all groups of rats in the liver and gastrocnemius muscle (data not shown).

### 3.5. Analysis of Hepatic Steatosis

**Figure 4:**
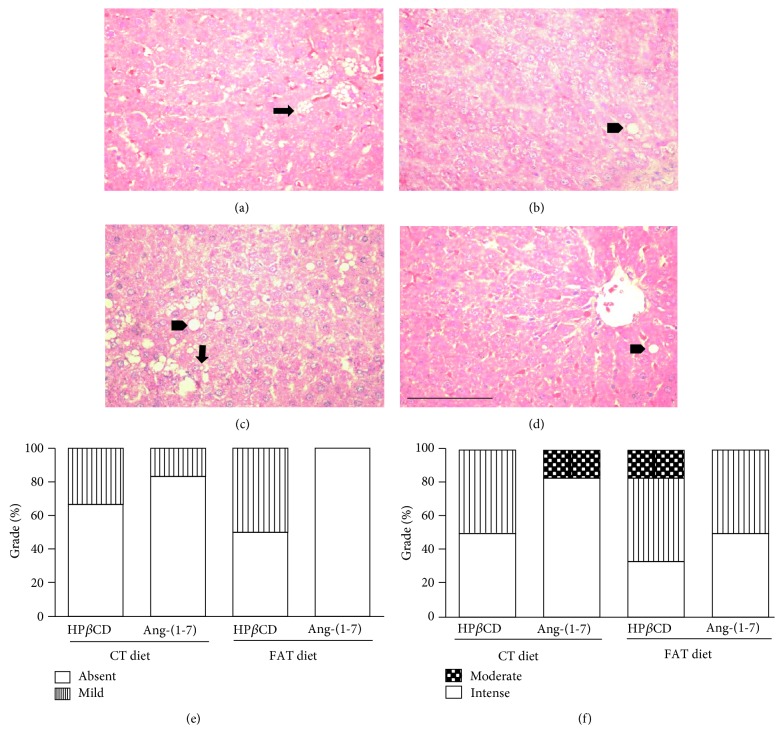
Photomicrographs of the liver stained with hematoxylin and eosin (HE). The arrow indicates microvesicular steatosis, and the arrowhead indicates macrovesicular steatosis. (a) Rats fed with control (CT) diet and treated only with the vehicle (HP*β*CD/empty). (b) Rats fed with CT diet and treated with HP*β*CD-Ang-(1-7). (c) Rats fed with high-fat (FAT) diet and treated with HP*β*CD/empty. (d) Rats fed with FAT diet and treated with HP*β*CD-Ang-(1-7). Magnification ×440. Bar = 50 *μ*m. Qualitative evaluation of macrovesicular steatosis (e) and microvesicular steatosis (f) in the hepatic tissue, using the degrees of Absent, Mild, Moderate, or Intense.

Liver histology revealed that FAT-HP*β*CD/empty rats presented macrovesicular steatosis with 50% of hepatocytes with mild grade, whereas FAT-HP*β*CD/Ang-(1-7) animals did not present macrovesicular steatosis (Figures 4(a)–4(e)). In relation to microvesicular steatosis, FAT-HP*β*CD/empty animals presented 50% of the hepatocytes with a mild grade and 16.6% of the hepatocytes with a moderate grade, while the FAT-HP*β*CD/Ang-(1-7) group had 50% of a discrete grade and 50% absence of hepatocytes (Figures 4(a), 4(d), and 4(f)).

## 4. Discussion

In the present study, we showed that the treatment with oral formulation of Ang-(1-7), HP*β*CD/Ang-(1-7), in the last six weeks in rats fed with FAT diet for 13 weeks was efficient in restoring RAS components and biometric (body mass and adiposity index) and biochemical (triglycerides, LDL, total cholesterol, fasting glucose, and ALT) parameters, reducing oxidative stress, and improving the insulin signaling pathway in the liver and gastrocnemius muscle and macrovesicular liver damage.

In previous studies [6, 11, 15] using the same diet protocol, we showed that rats fed with FAT diet for 7 weeks had disorders related to human MetS, such as increased fat deposits (epididymal, retroperitoneal, and inguinal), MAP, HR, fasting glucose, ALT, and total cholesterol levels. In the present study on the seventh week, the FAT-HP*β*CD/empty rats already had disorders characteristic of MetS such as elevated fasting glucose, body mass, MAP, and HR, so the present study refers to treatment and not prevention to the development of MetS.

This present study showed that oral Ang-(1-7) treatment in established MetS rats normalized body mass, adiposity index, LDL cholesterol, and triglycerides compared to untreated MetS rats. These data are in agreement with studies of prevention in transgenic rat model of inducible DM2 (Tet29) [18] insulin resistance and mice (FVB/N) fed with FAT diet [4] that oral Ang-(1-7) induced reductions on fat deposits, total cholesterol, ALT levels [4], and glucose levels [18].

Studies have shown that the ACE/Ang II/AT1 receptor axis is involved with oxidative stress and IR in the DM2 and MetS states [7, 8, 18–20]. The unbalance between reactive oxygen species (ROS) and detoxification by antioxidant enzymes can affect intracellular signaling pathways being an important mechanism for metabolic diseases [5, 21]. Oxidative damage is related to IR in the liver and gastrocnemius muscle [21, 22]. In addition, the hepatic CAT enzyme activity that is responsible for the part of H_2_O_2_ elimination is reduced in the liver in situations of MetS in the rat model [6, 21]. It has already been established in the literature that Ang II participates in these cardiometabolic diseases due to its pro-oxidant actions, while Ang-(1-7) presents regulatory actions against Ang II actions [1, 20, 23, 24]. In fact, Cao et al.'s study [12] showed in knockout mice for ACE2 (ACE2 KO) presented IR and elevated levels of ROS in the liver. Additionally, in the prevention study in FVB/N mice fed with FAT diet [19] or in high-fructose obese mouse model [25], Ang-(1-7) improved superoxide production in epididymal fat and improved lipid metabolism associated with increased *Ace2* and decreased *Ace* expression in the liver [25]. The present study is in agreement with these studies, where FAT-HP*β*CD/empty rats showed increased expression of angiotensinogen, *ACE*, and *AT1R* in the liver, increased TBARS concentration, and decreased CAT activity in the liver and in the gastrocnemius muscle. The oral treatment with Ang-(1-7) was effective in decreasing hepatic *ACE* and *AT1R*, increasing *ACE2* gene expression, and restoring CAT activity and TBARS concentration in FAT-HP*β*CD/Ang-(1-7) rats. In addition, our data showed that Ang-(1-7) treatment increased the SOD activity in the liver, thus increasing the ability to dismutase the superoxide anion to H_2_O_2_ and reducing the oxidation products by reducing TBARS concentration in FAT-HP*β*CD/Ang-(1-7) rats.

In prevention studies, using oral administration of HP*β*CD/Ang-(1-7) in transgenic DM2 mice [18] and FVB/N mice fed with FAT diet [4] showed, in epididymal adipose tissue, normalization of glucose tolerance, insulin sensitivity, and fasting blood glucose [4, 18]. Additionally, Giani et al.'s and Muñoz et al.'s studies [10, 24] have showed in rats fed with 10% fructose in water for six weeks and treated with Ang-(1-7) by osmotic pump in the last two weeks of diet the normalization of insulin signaling components (IR/IRS-1/PI3K/AKT) in the skeletal muscle, liver, and adipose tissue. Accordingly, the present data showed a reduction in mRNA expression of IRS-1 and AKT-2 in the liver and gastrocnemius muscle while GLUT-4 was reduced only in gastrocnemius muscle in FAT-HP*β*CD/empty rats. However, the treatment with Ang-(1-7) in the last six weeks of FAT diet became the expression of AKT-2 and GLUT-4 in the liver and in the gastrocnemius muscle and the expression of IRS-1 in the liver of FAT-HP*β*CD/Ang-(1-7) rats similar to CT-HP*β*CD/empty rats.

Oxidative stress plays an important role in decreasing insulin sensitivity in the liver. EROs are produced by many processes [9], and in skeletal muscle cells, the activation of NADPH oxidase induced by Ang II may worsen insulin signaling [26], 2006). The ACE2/Ang-(1-7)/Mas axis, that counterregulating effects of the ACE/Ang II/AT1R axis, is involved in the reduction of insulin resistance through antioxidant effects [8, 12]. Cao et al. [12] demonstrated in HepG2 cells that Ang-(1-7) protects against oxidative stress by inhibiting the expression of NAPDH oxidase contributing to the increasing levels of ROS that can lead to the activation of N-terminal c-Jun kinases (JNKs), which can phosphorylate IRS proteins [27]. Cao et al. [12] showed that overexpression of ACE inhibited JNK phosphorylation, leading to decreased phosphorylation of the IRS-1 serine residue, which increased glucose uptake in HepG2 cells. In addition, Ang-(1-7) increases the protein expression of GLUT-4 in the skeletal muscle of ACE2 KO mice [27] and induces the phosphorylation of kinase that participates in the translocation of GLUT-4, PI3K-C2alpha, in human endothelial cells [28] in response to insulin stimulation [24, 29]. Accordingly, data from the present study show that Ang-(1-7) treatment, despite not reversing the expression of all evaluated components of the insulin pathway, made the expression of IRS-1, AKT-2, and GLUT-4 in the liver and AKT-2 and GLUT-4 in the muscle similar to the levels of expression of the control rats (HP*β*CD/empty). Additionally, Williams et al. [30] showed that adult male C57BL/6J mice receiving a high lipid diet for 11 weeks and treated with Ang-(1-7) infused during the last 3 weeks of diet showed increased GLUT-4 in the skeletal muscle that were sufficient to improve insulin resistance and glucose uptake. All these data together suggest that the beneficial effect of Ang-(1-7) on improving the insulin signaling pathway is probably due to restoring RAS components and redox balance in the liver and gastrocnemius muscle in MetS rats.

Hepatic IR correlates with increased fat and steatosis in the liver [3, 6, 11]. In our present study, there is increased hepatic fat accumulation in FAT-HP*β*CD/empty rats which was attenuated by treatment with HP*β*CD/Ang-(1-7). These data are in accordance with the prevention study of Feltenberger et al. [19] in FVB/N mice fed with FAT diet in which HP*β*CD/Ang-(1-7) reduced total weight and steatosis in the liver. Our hypothesis, based on the present data, is that the composition of FAT diet increased the circulation of free fatty acids, which could be captured and undergo lipid peroxidation products by the ROS leading to a process of hepatic steatosis, probably by *Ace* and *AT1R* contribution [2, 3, 21]. Furthermore, the steatosis may be related to hepatic IR resulting from cell injury or hepatic inflammation [19]. In the present study, the treatment with HP*β*CD/Ang-(1-7) was effective in restoring this damage in the liver of FAT-HP*β*CD/Ang-(1-7) rats by normalizing body mass, adiposity index, LDL cholesterol, and triglycerides; decreasing *ACE* and *AT1R*; increasing *Ace2* gene expression; and reversing lipid peroxidation in the liver and gastrocnemius muscle.

In conclusion, our data in rats with stablished MetS-induced FAT diet for 13 weeks and orally treated with HP*β*CD/Ang-(1-7) in the last six weeks show that Ang-(1-7) was efficient in normalizing lipid metabolism, improving the insulin signaling pathway in the liver and gastrocnemius muscle, function, and macrovesicular liver damage, probably by restoring the unbalance between RAS axis and reducing oxidative stress in the liver and gastrocnemius muscle. This protective effect of Ang-(1-7) in the liver and muscle can probably contribute in reversing the hyperglycemia and dyslipidemia in MetS-induced FAT diet rats.

## Figures and Tables

**Table 1:** Evaluation of MetS establishment in rats after 7 weeks of FAT diet.

Parameters	CT-HP*β*CD/empty	FAT-HP*β*CD/empty
Body mass (g)	168 ± 6.7	275 ± 9.1^∗^
Blood glucose level (mg/dL)	101 ± 1.6	127 ± 2.5^∗^
MAP (mmHg)	106 ± 4.5	136 ± 2.0^∗^
HR (bpm)	368 ± 6.3	433 ± 6.3^∗^
*N*	6-14	6-14

^∗^ *p* < 0.05 compared to CT-HP*β*CD/empty group. Values are expressed as mean values ± standard error of the mean and analyzed using unpaired student *t-*test. MAP = mean arterial pressure; HR = heart rate; *N* = number of animals.

**Table 2:** Nutritional, biometric, and biochemical parameters of rats fed with a control diet or high-fat diet for 13 weeks and treated with HP*β*CD or HP*β*CD/Ang-(1-7) during the last 6 weeks of the diet.

Parameters	Experimental groups			
	CT diet		FAT diet	
	HP*β*CD	HP*β*CD/Ang-(1-7)	HP*β*CD	HP*β*CD/Ang-(1-7)
Food intake (g)	87.5 ± 5.3	84.4 ± 1.5	72.5 ± 3.9^∗^	72.3 ± 1.2^∗^
Caloric intake (kcal)	332.4 ± 20.2	320.8 ± 5.5	377.2 ± 20.6	376.2 ± 6.5
Liver (g/100 g rat mass)	2.73 ± 0.04	2.71 ± 0.07	2.8 ± 0.11	2.57 ± 0.04
Gastrocnemius (g/100 g rat mass)	1.31 ± 0.03	1.28 ± 0.03	1.15 ± 0.08	1.24 ± 0.02
Adiposity index	6.7 ± 0.4	5.3 ± 0.4	10.7 ± 0.2^∗^	8.2 ± 0.6^#^
Body mass (g)	282.7 ± 2.2	273.9 ± 13.9	348.7 ± 9.4^∗^	259.6 ± 9.1^#^
Fasting glucose (mg/dL)	111 ± 1.4	113 ± 1.95	123.1 ± 1.7^∗^	117.4 ± 1.9
Total cholesterol (mg/dL)	65.6 ± 1.6	64.4 ± 1.3	75.1 ± 2.5^∗^	70.3 ± 1.9
LDL cholesterol (mg/dL)	9.4 ± 2.0	10.2 ± 1.4	27.3 ± 3.3^∗^	26.5 ± 3.3^#^
HDL cholesterol (mg/dL)	30.5 ± 0.9	29.12 ± 0.6	28.4 ± 1.3	29.8 ± 1.1
Triglycerides (mg/dL)	42.1 ± 3.5	38.9 ± 3.6	68.2 ± 5.9^∗^	40.7 ± 4.5^#^
ALT (U/L)	52.7 ± 0.8	52.1 ± 8.6	70.9 ± 2.1^∗^	60.7 ± 1.9
AST (U/L)	3.5 ± 0.8	2.7 ± 0.2	2.9 ± 0.6	3.2 ± 1.4
*N*	7	7	7	7

^∗^ *p* < 0.05 compared to CT-HP*β*CD/empty group; ^#^*p* < 0.05 compared to HF-HP*β*CD/empty group (two-way ANOVA followed by Bonferroni test), expressed as mean values ± standard error of the mean. *N* = number of animals.

## Data Availability

The [DATA TYPE] data used to support the findings of this study are included within the article.
